# Primary hemostatic function in dogs with acute kidney injury

**DOI:** 10.1111/jvim.15588

**Published:** 2019-08-05

**Authors:** Duana McBride, Rosanne E. Jepson, Stefano Cortellini, Daniel L. Chan

**Affiliations:** ^1^ Department of Clinical Science and Services, The Royal Veterinary College University of London London United Kingdom

**Keywords:** buccal mucosal bleeding time, platelet aggregometry, renal, thrombocytopathia, von Willebrand factor

## Abstract

**Background:**

Bleeding tendencies can occur with uremia.

**Objectives:**

To characterize primary hemostatic function in dogs with acute kidney injury (AKI).

**Animals:**

Ten dogs with International Renal Interest Society AKI grade III or above and 10 healthy controls.

**Methods:**

Prospective study comparing PCV, platelet count, platelet aggregometry (Multiplate), and von Willebrand factor antigen to collagen binding activity ratio (vWF:Ag:vWF:CBA) in 2 groups of dogs (AKI group versus controls). Buccal mucosal bleeding time was measured in the AKI group only. Data are presented as median [25th, 75th percentile] unless otherwise stated. Significance was set at *P* < .05.

**Results:**

Mean PCV was significantly lower in the AKI (34.7%; ±SD, 8.8) than in the control (46.1%; ±SD, 3.6; *P* < .001) group. Platelet count was significantly higher in the AKI (350.5 × 10^3^/μL [301, 516]) than in the control (241 × 10^3^/μL [227, 251]; *P* = .01) group. Collagen‐activated platelet aggregometry measured as area under the curve was significantly lower in the AKI (36.9 ± 17.7) than in the control (54.9 ± 11.2; *P* = .05) group. vWF:Ag:vWF:CBA was significantly higher in the AKI (2.2 [1.9, 2.6]) than in the control (1.1 [1.1, 1.2]; *P* = .01) group. There was a strong correlation between vWF:Ag:vWF:CBA and creatinine (*r* = 0.859; *P* < .001), but no other variables.

**Conclusions and Clinical Importance:**

Dogs with AKI had decreased collagen‐activated platelet aggregation and appear to have a type II von Willebrand disease‐like phenotype as indicated by the high vWF:Ag:vWF:CBA.

**Abbreviations**
AA: arachidonic acid
ADP: adenosine diphosphate
AKI: acute kidney injury
AoCKD: acute‐on‐chronic kidney disease
AUC: area under the curve
AVWS: acquired von Willebrand syndrome
BMBT: buccal mucosal bleeding time
CKD: chronic kidney disease
COL: collagen
GP: glycoprotein
IRIS: International Renal Interest Society
LTA: light transmittance aggregometry
MEPA: multiple electrode impedance platelet aggregometry
MW: molecular weight
PRP: platelet‐rich plasma
SD: standard deviation
TP: total protein
vWF: von Willebrand Factor
vWF:Ag: von Willebrand factor antigen
vWF:CBA: von Willebrand factor collagen binding activity
vWF:RCo: von Willebrand factor ristocetin
vWFAg:CBA: von Willebrand factor antigen to collagen binding activity ratio

## 1 INTRODUCTION

Acute kidney injury (AKI) represents a spectrum of acute diseases, encompassing a continuum of functional and parenchymal damage. The resultant renal injury will depend on the functional origin, severity, and duration of the conditions inciting kidney injury (www.iris-kidney.com/guidelines). Dogs with AKI might require invasive procedures such as renal biopsies for diagnosis, prognostication, and treatment.^1^ Other invasive procedures such as placement of central venous catheters, dialysis catheters for renal replacement treatment, peritoneal catheters for peritoneal dialysis,^2^, ^3^ or feeding tube placement for longer term management might also be required.^4^

Uremic bleeding occurs in people with chronic kidney disease (CKD) due to abnormalities in primary hemostasis.^5^, ^6^, ^7^ Intrinsic factors including platelet secretion defects, abnormal platelet‐vessel wall interactions including von Willebrand Factor (vWF) abnormalities, increased platelet inhibitors, uremic toxins, as well as factors such as anemia, if present, contribute to bleeding.^5^, ^6^, ^7^ Extrinsic factors might also be involved, including medications, comorbidities, and iatrogenic factors such as extracorporeal circulation.^5^, ^6^, ^7^ Although these mechanisms are well described in people with CKD, the understanding of uremic bleeding in dogs with AKI is limited.^8^, ^9^

It is known that platelet function is altered in uremic dogs; however, the mechanisms are not clearly understood. In experimental models of kidney injury, prolongation in buccal mucosal bleeding time (BMBT) and decreased platelet glass bead retention percentage occurs without significant changes in arachidonic acid (AA)‐, adenosine diphosphate (ADP)‐, collagen (COL)‐, and epinephrine‐induced light transmittance aggregometry (LTA).^10^ In addition, vWF antigen concentrations (vWF:Ag) increases,^11^ suggesting 1 of the mechanisms of bleeding to be due to abnormalities in platelet adhesion independent of vWF or platelet agonists. In dogs with naturally occurring CKD, significant changes in AA‐, ADP‐, and COL‐induced multiple electrode impedance platelet aggregometry (MEPA) does not occur despite clinical bleeding.^12^ However, platelet dysfunction as measured by the platelet function analyzer‐100 does occur.^13^

Despite wide acceptance that platelet dysfunction occurs in people with CKD,^5^, ^6^, ^7^ there is not a comprehensive understanding of hemostatic defects in dogs with naturally occurring AKI. Therefore, the primary objective of this study was to characterize primary hemostatic function measured by AA‐, ADP‐, and COL‐induced MEPA, and vWF assays in dogs with naturally occurring AKI. The secondary objective was to determine if BMBT can be used to predict primary hemostatic dysfunction in dogs with AKI when compared to MEPA and vWF assays. We hypothesized that (1) there will be no difference in AA‐, ADP‐, and COL‐induced MEPA between dogs with AKI and healthy dogs; (2) there will be no difference in vWF:Ag, vWF collagen binding activity (vWF:CBA), and vWF:Ag to vWF:CBA ratio (vWF:Ag:vWF:CBA) between dogs with AKI and healthy dogs; and (3) there will be strong correlation between BMBT and these primary hemostatic variables measured.

## 2 MATERIALS AND METHODS

This prospective observational cohort study was approved by the Ethics and Animal Welfare Committee of the Royal Veterinary College (reference number 2014 1305), with owner consent obtained for all dogs enrolled. Client‐owned dogs admitted to the Queen Mother Hospital for Animals of the Royal Veterinary College between June 2015 and July 2016 with International Renal Interest Society (IRIS) AKI grade III or higher were enrolled as the AKI group. Dogs were assigned an IRIS AKI grade III based on a serum creatinine concentration ≥2.6 mg/dL (www.iris-kidney.com/guidelines/grading); an acute onset of clinical signs attributable to AKI including listlessness, vomiting, diarrhea, and anorexia; exclusion of prerenal causes of azotemia by documentation of isosthenuria (urine specific gravity of 1.007 to 1.015) before IV fluid therapy (excluding other causes of isosthenuria); azotemia in the presence of euvolemia or hypervolemia based on clinical assessment; and exclusion of post‐renal causes based on the attending clinicians assessment. Dogs with acute‐on‐chronic kidney disease (AoCKD) were included in the study. Chronic kidney disease was diagnosed based on the presence of 1 of more of the following: (1) history of polyuria and polydipsia, (2) being previously diagnosed with kidney disease, (3) presence of nonregenerative anemia with no evidence of recent hemorrhage, (4) ultrasonographical changes supportive of CKD reported by a board‐certified veterinary radiologist, and (5) histological diagnosis reported by a board‐certified anatomical pathologist. A diagnosis of AoCKD was made based on acute onset of clinical signs attributable to azotemia with evidence of CKD. Dogs were excluded from the study if they weighed <3.5 kg; received anticoagulants, starch‐based colloids, antiplatelet, or nonsteroidal anti‐inflammatory drug treatment during the preceding 14 days; were thrombocytopenic with a platelet count of <100 × 10^3^/μL; were anemic with a PCV of <20%; had a diagnosis of sepsis including pyelonephritis but not excluding leptospirosis; were of a breed with a hereditary predisposition to platelet dysfunction including Greyhounds, Cavalier King Charles Spaniels, and Doberman Pinschers; or if minimum database including CBC (Advia 2120i, Siemens, UK) with platelet count confirmed by a board‐certified clinical pathologist, biochemistry (iLAB 600; Werfen, New Delhi, India) with urea and creatinine measurements, urinalysis, and urine culture and specificity were not obtained.

Healthy dogs were enrolled as the control group from the hospital blood donor program. The number of dogs in the control group matched the number of dogs in the AKI group. All dogs were between 1 and 8 years of age, with body weight greater than 25 kg; had not received any medication during the preceding 14 days; and were deemed healthy based on physical examination, CBC (Advia 2120i, Siemens), and serum biochemistry (iLAB 600, Werfen). Any dogs within the blood donor program with a breed predisposition to platelet dysfunction including Greyhounds and Doberman Pinschers were excluded from the study.

Once a diagnosis of AKI was made, client consent for study enrolment was obtained, and diagnostic tests and treatment were performed at the discretion of the attending clinician. Age, breed, sex, and minimum database as described above were collected before or during the study, and additional information including evidence of clinical bleeding and final diagnosis was obtained from medical records after the study was concluded. When routine blood sampling was required at the discretion of the attending clinician, a 21‐gauge blood collection system (BD Safety‐Lok, Becton Dickinson, UK) was inserted in the jugular or saphenous vein, and 1.8 mL of blood was collected in a 3.2% sodium citrate anticoagulated vacutainer tube (BD Vacutainer, Becton Dickinson), 1.5 mL of blood was collected in a hirudin anticoagulated vacutainer tube (Roche Diagnostic International Limited, Rotkreuz, Switzerland), and remaining blood samples were collected at the discretion of the attending clinician. Dogs in the control group had a 21‐gauge blood collection system (BD Safety‐Lok) inserted in the saphenous vein, and 3 mL of blood collected in an ethylenediaminetetraacetic acid anticoagulated vacutainer tube (Cell‐Dyn 2500; Abbott Diagnostics, Abbott Park, Illinois) and 3.5 mL of blood collected in a silica‐coated vacutainer tube (ILAB 600, Holliston, Massachusetts) for routine hematology and biochemistry, respectively, followed by 1.8 mL of blood collected in a 3.2% sodium citrate anticoagulated vacutainer tube (BD Vacutainer), and 1.5 mL of blood collected in a hirudin anticoagulated vacutainer tube (Roche Diagnostic International Limited). The hirudin anticoagulated blood was always collected as the second or subsequent sample in both groups to prevent venepuncture‐induced platelet activation.

Arachidonic acid (ASPItest, Roche Diagnostic International Limited), ADP (ADPtest, Roche Diagnostic International Limited), and COL‐activated (COLtest, Roche Diagnostic International Limited) MEPA (Multiplate) (Roche Diagnostic International Limited) were performed on the hirudin anticoagulated blood samples according to a previously published protocol within 30 to 120 minutes of sample collection.^14^ Changes in electrical impedance were measured, and area under the curve (AUC) of aggregation units over a 6‐minute interval was reported. Area under the curve of aggregation units was measured in duplicate with results reported as the mean of the duplicate measurements. Measurements were repeated if Pearson's correlation coefficient and the difference between the 2 areas under the curve were less than 0.98 or greater than 20%, respectively. Reference intervals for MEPA were generated from sampling of 34 blood donors that were considered healthy based on history, clinical examination, hematology, and biochemistry. As the sample size was small, Horn's method was used for outlier detection and robust method was used for the calculation of reference intervals.^15^ This was implemented using refLimit function from <referenceIntervals> package in the R software (R Studio, version 1.1.456, Boston, Massachusetts).

Sodium citrate anticoagulated blood was centrifuged at 2500*g* for 20 minutes at 4°C within 30 minutes of sample collection. After centrifugation, plasma was collected and stored at −80°C for no longer than 6 months. Von Willebrand Factor antigen concentrations, vWF:CBA, and vWF:Ag:vWF:CBA were measured from the citrated plasma by an external laboratory as previously described.^16^, ^17^ Reference interval for these values was developed by the same external laboratory (Cornell University Animal Health Diagnostic Centre, Ithaca, New York).

Buccal mucosal bleeding time was performed only in the AKI group, and this occurred within 2 hours of aforementioned blood sample collection. This procedure was performed according to published protocols,^18^, ^19^ by 2 primary investigators. If bleeding from the incision site continued beyond 10 minutes, BMBT was discontinued by applying digital hemostasis until bleeding discontinued.

Shapiro–Wilk test for normality was performed on age, body weight, AA AUC, ADP AUC, COL AUC, vWF:Ag, vWF:CBA, wVF:Ag:vWF:CBA, PCV, platelet count, creatinine, and urea with *P* > .05 being normally distributed. Of the data that were normally distributed, Levene's test for equality of variance was performed in which equal variance was assumed if *P* > .05. Student's *t*‐test was performed to compare the magnitude of difference if the data were normally distributed and equal variance assumed; otherwise, Mann–Whitney *U* test was performed with *P* < .05 being significantly different. Spearman's rank was used to measure correlations between all the above‐mentioned continuous variables in addition to BMBT. Statistical analyses were performed using commercially available software (IBM SPSS Statistics for Windows, version 25.0, Armonk, New York).

## 3 RESULTS

During the recruitment period, 58 dogs presented with AKI, and from these, 10 dogs met the inclusion criteria for the AKI group and 10 control dogs were enrolled to match this number. Within the AKI group, 2 were male entire, 4 were male neutered, and 4 were female neutered. Within the control group, 4 were male entire, 4 were male neutered, 1 was female entire, and 1 was female neutered. Breeds in the AKI group included a German Shepherd Dog cross, Jack Russell Terrier, Cairn Terrier, Labrador Retriever, Golden Retriever, Border Collie, Boston Terrier, English Springer Spaniel, and 2 crossbreed dogs. Breeds included in the control group included 4 Labrador Retrievers, 2 German Shepherd Dogs, 2 German Shepherd Dog crosses, 1 Boxer, and 1 Pyrenean Mountain Dog. There were significant differences in age and body weight between the 2 groups (Table 1).

**Table 1:** Measured variables in the acute kidney injury (AKI) and control groups

Variables	Reference intervals	AKI group	Control group	*P* value
Age (y)		9.7 (±2.9)	4.3 (±1.9)	.01*
Body weight (kg)		15.1 [6.1, 37.4]	36.20 [29.0, 48.6]	.01*
PCV (%)	37‐55	34.7 (±8.8)	46.1 (±3.6)	.001*
Plt (×10^3^/μL)	177‐398	350 [301, 516]	241.00 [227, 251]	.01*
Creatinine (g/dL)	0.7‐1.8	8.5 (±4.3)	1.2 (±0.2)	<.001*
Urea (mg/dL)	5‐30	135.1 (±62.4)	6.5 (±1.01)	<.001*
AA AUC	13.6‐74.9	28.4 (±22.0)	42.3 (±7.5)	.14
ADP AUC	10.1‐103.7	46.0 [21.5, 64.3]	60.50 [46.5, 62.0]	.70
COL AUC	6.2‐105.3	36.9 (±17.7)	54.9 (±11.2)	.05*
vWF:Ag (%)	70‐180	125.6 (±28.1)	104.7 (±39.4)	.08
vWF:CBA	50‐170	55.0 (±16.1)	88.7 (±26.9)	.04*
vWF:Ag:vWF:CBA	0.5‐1.7	2.2 [1.9, 2.6]	1.1 [1.1, 1.2]	.001*

All variables are reported as mean (±SD), other than body weight, platelet count, and ADP AUC, which are reported as median [25th, 75th percentile].

Abbreviations: AA, arachidonic acid; ADP, adenosine diphosphate; AUC, area under the curve; COL, collagen; Plt, platelet count; vWF:Ag, von Willebrand factor antigen; vWF:CBA, von Willebrand factor collagen binding activity; vWF:Ag:vWF:CBA, von Willebrand factor antigen to von Willebrand factor collagen binding activity ratio.

* *P* < .05 was considered significant.

The final diagnosis of AKI included 5 dogs with AoCKD for which a reason for the acute deterioration was not identified, and 1 dog with AKI secondary to each of the following diagnoses: hypertensive nephropathy secondary to pheochromocytoma, leptospirosis, cutaneous and renal glomerular vasculopathy, pancreatitis, and gentamicin administration with associated pancreatitis.

Invasive procedures were performed in 6 dogs in the AKI group, including nasoesophageal tube placement, esophagostomy tube placement, renal biopsy, bone marrow biopsy, and prostatic wash with no evidence of clinical bleeding. Three dogs had gastrointestinal hemorrhage; however, the underlying cause was not determined. One dog was anemic at the time of admission with a PCV and total protein (TP) of 28% and 4.3 g/dL, respectively, with no clinical bleeding despite nasoesophageal tube placement. One dog was anemic with a PCV and TP of 14% and 4.8 g/dL, respectively, at the time of hospital admission with no clinical sign of bleeding despite esophagostomy tube placement. This dog was included in the study after receiving a blood transfusion. Two dogs had decreasing PCV with 1 requiring 2 blood transfusions after the study period, despite no evidence of clinical bleeding.

Packed cell volume (*P* = .001), COL AUC (*P* = .05), and vWF:CBA (*P* = .04) were significantly lower in the AKI group compared to the control group, although COL AUC remained within reference interval. Platelet count (*P* = .01), creatinine (*P* < .001), urea (*P* < .001), and vWF:Ag:vWF:CBA (*P* = .001) were significantly higher in the AKI group compared to the control group. There was no significant difference between the 2 groups in other measured variables (Table 1). There was no significant difference in any variable between the dogs with AoCKD and dogs with no underlying CKD (data not presented).

Buccal mucosal bleeding time was performed on 9 of the 10 dogs in the AKI group. One dog did not have BMBT performed due to clinical concerns that the BMBT procedure might result in worsening anemia due to the small size of the dog and low MEPA AUC. The mean and standard deviation (SD) of BMBT in dogs with AKI was 5.09 (±2.68) minutes (reference interval, 1.8‐4.8 minutes^18^).

There was a strong correlation between creatinine and age (*r* = 0.721, *P* < .001); creatinine and vWFAg:CBA (*r* = 0.859, *P* < .001); ADP and COL (*r* = 0.745, *P* < .001); urea and platelet count (*r* = 0.746, *P* < .001); and urea and creatinine (*r* = 0.716, *P* < .001). There was moderate correlation between creatinine and PCV (*r* = −0.628, *P* = .003); creatinine and vWF:CBA (*r* = −0.621, *P* = .004); vWF:Ag:vWF:CBA and PCV (*r* = −0.519, *P* = .02); vWF:Ag:vWF:CBA and vWF:Ag (*r* = 0.506, *P* = .02); vWF:Ag:vWF:CBA and vWF:CBA (*r* = −0.542, *P* = .01); vWF:Ag:vWF:CBA and age (*r* = 0.583, *P* = .007); vWF:CBA and age (*r* = −0.636, *P* = .003); AA AUC and PCV (*r* = 0.535, *P* = .02); PCV and age (*r* = −0.506, *P* = .02); urea and age (*r* = −0.628, *P* = .001); urea and vWF:CBA (*r* = −0.617, *P* = .004); urea and wVF:Ag:vWF:CBA (*r* = 0.642, *P* = .002); and with urea and PCV (*r* = −0.522, *P* = .02). There was no significant correlation between other variables (Table 2).

**Table 2:** Spearman's rank correlation coefficient (*r*) between all measured variables from acute kidney injury and control groups

		Age	BW	PCV	Plt	Crea	Urea	AA AUC	ADP AUC	COL AUC	vWF:Ag	vWF:CBA	vWF:Ag:vWF:CBA	BMBT
Age	*r*	1.000	−0.392	−0.506*	0.414	0.721**	0.686*	−0.219	−0.178	−0.468	−0.077	−0.636*	0.583*	−0.167
	*P* value	…	.09	.02	.09	<.001	.001	.35	.45	.04	.75	.003	.007	.67
BW	*r*	−0.392	1.000	0.494	−0.352	−0.450	−0.451	0.138	−0.033	0.047	−0.111	0.195	−0.235	0.317
	*P* value	.09	…	.03	.15	.05	.05	.56	.89	.84	.64	.41	.32	.41
PCV	*r*	−0.506*	0.494	1.000	−0.313	−0.628*	−0.522*	0.535*	−0.169	0.115	−0.145	0.467	−0.519*	0.333
	*P* value	.02	.03	…	.21	.003	.02	.02	.48	.63	.54	.04	.02	.38
Plt	*r*	0.414	−0.352	−0.313	1.000	0.318	0.746**	0.068	0.071	−0.249	0.085	−0.212	0.197	−0.286
	*P* value	.09	.15	.21	…	.20	<.001	.79	.78	.32	.74	.40	.43	.54
Crea	*r*	0.72**	−0.450	−0.628*	0.318	1.000	0.716**	−0.146	0.008	−0.430	0.265	−0.621*	0.859**	0.167
	*P* value	<.001	.05	.003	.20	…	<.001	.54	.98	.06	.26	.004	<.001	.67
Urea	*r*	0.686*	−0.451	−0.522*	0.746**	0.716**	1.000	−0.189	−0.072	−0.432	0.093	−0.617*	0.642*	0.250
	*P* value	.001	.05	.02	<.001	<.001	…	.42	.76	.06	.70	.004	.002	.52
AA AUC	*r*	−0.219	0.138	0.535*	0.068	−0.146	−0.189	1.000	0.155	0.240	−0.055	0.142	−0.190	−0.250
	*P* value	.35	.56	.02	.79	.54	.42	…	.54	.31	.82	.55	.42	.52
ADP AUC	*r*	−0.178	−0.033	−0.169	0.071	0.008	−0.072	0.155	1.000	0.745**	0.157	0.122	−0.033	−0.400
	*P* value	.45	.89	.48	.78	.98	.76	.51	…	<.001	.51	.61	.89	.29
COL AUC	*r*	−0.468	0.047	0.115	−0.249	−0.430	−0.432	0.240	0.745**	1.000	−0.100	0.336	−0.488	−0.326
	*P* value	.04	.84	.63	.32	.06	.06	.31	<.001	…	.67	.15	.03	.39
vWF:Ag	*r*	−0.077	−0.111	−0.145	0.085	0.265	0.093	−0.055	0.157	−0.100	1.000	0.421	0.506*	−0.550
	*P* value	.75	.64	.54	.74	.26	.70	.82	.51	.67	…	.06	.02	.12
vWF:CBA	*r*	−0.636*	0.195	0.467	−0.212	−0.621*	−0.617*	0.142	0.122	0.336	0.421	1.000	−0.542*	−0.400
	*P* value	.003	.41	.04	.40	.004	.004	.55	.61	.15	.06	…	.01	.29
vWF:Ag:vWF:CBA	*r*	0.583*	−0.235	−0.519*	0.197	0.859**	0.642*	−0.190	−0.033	−0.488	0.506*	−0.542*	1.000	0.017
	*P* value	.007	.32	.02	.43	<.001	.002	.42	.89	.03	.02	.01	…	.96
BMBT	*r*	−0.167	0.317	0.333	−0.286	0.167	0.250	−0.250	−0.400	−0.326	−0.550	−0.400	0.017	1.000
	*P* value	.67	.41	.38	.54	.67	.52	.52	.29	.39	.12	.29	.96	…

Abbreviations: AA, arachidonic acid; ADP, adenosine diphosphate; AUC, area under the curve; BMBT, buccal mucosal bleeding time; BW, body weight; COL, collagen; Crea, creatinine; Plt, platelet count; vWF Ag, von Willebrand factor antigen; vWF:Ag:vWF:CBA, von Willebrand factor antigen to collagen binding activity ratio; vWF CBA, von Willebrand factor collagen binding activity.

* *r* > ±0.5 and *P* < .05.

** *r* > ±0.7 and *P* < .05.

## 4 DISCUSSION

This study assessing primary hemostatic function in dogs with AKI found that COL‐, but not AA‐ or ADP‐, induced platelet aggregation as measured by MEPA was significantly decreased in dogs with AKI compared to a control population of healthy dogs; however, aggregation remained within reference intervals. Although there was no difference in vWF:Ag concentration, vWF:Ag:vWF:CBA was significantly higher in dogs with AKI, indicating that less vWF was bound to COL possibly due to a reduction in higher molecular weight (MW) vWF multimers in dogs with AKI. This was supported by the correlation between vWF:Ag:vWF:CBA and both creatinine and urea. Although BMBT was marginally prolonged in dogs with AKI, there was no correlation between BMBT and any other variables. However, these aforementioned results must be interpreted in light of the difference in the lower PCV and higher platelet count in the dogs with AKI.

Our study suggests that COL‐inducted platelet aggregation is impaired in dogs with AKI. Collagen found in vessel walls and basement membranes are important in normal platelet function as it directly binds to glycoprotein (GP) VI receptors and integrin α_2_β_1_, and indirectly via vWF binding to GPIb‐IX‐V complex which are integral processes for normal platelet function.^20^ Dogs with CKD also have impairment in COL‐induced platelet function as measured by platelet function analyzer's COL‐ and ADP‐induced platelet closure time.^13^ However AA‐, ADP‐, or COL‐induced platelet aggregation as measured by LTA are normal in dogs with CKD^12^ and experimentally induced kidney injury.^10^ One case report describes a dog with CKD with decreased AA‐ and ADP‐induced LTA; however, COL‐induced aggregometry was not performed.^21^ The difference in results might be explained by the different nature of kidney injury and the difference in methodology of testing platelet function.

There are differences between MEPA and LTA. Light transmittance aggregometry has been traditionally considered the gold standard of platelet function tests,^22^ and like MEPA, platelet aggregation is measured after activating platelets with agonists including AA, ADP, and COL. The advantages of MEPA, however, are that it is a point‐of‐care test which utilizes whole blood, as opposed to platelet‐rich plasma (PRP) which is required for LTA, minimizing preanalytical and analytical variables. The use of whole blood reduces the time to analysis, obliterates the need to produce PRP in which platelets could be activated in the process, and the sample used is more physiological having erythrocytes present which augments platelet function by increasing AA release and expression of α_IIb_β_3_ receptors.^20^ Multiple electrode impedance platelet aggregometry has also been validated^23^, ^24^ and used in dogs to assess platelet function in the setting of sepsis^14^ and hemorrhagic shock.^25^

When assessing platelet function, erythrocyte concentration must be taken into consideration, as it affects platelet function by 1 or more mechanisms. Erythrocytes release ADP and thromboxane which augment platelet activation and aggregation and scavenge nitric oxide which is a platelet antagonist.^26^ Anemia results in decrease in AA‐ and ADP‐induced, but not COL‐induced, platelet aggregation in dogs^25^ and in people.^27^, ^28^ Therefore, it is unlikely that the lower PCV in the AKI group would have influenced the decrease in COL‐ induced platelet aggregation in this study. There is positive linear correlation between platelet count and platelet aggregation measured by MEPA.^29^, ^30^, ^31^, ^32^ Therefore, it is possible that a type‐II error might have occurred with AA‐ and ADP‐induced platelet aggregation due to the significantly higher platelet count in the AKI group in the current study.

Although it is known that primary hemostatic dysfunction occurs in dogs with CKD, it is important to differentiate AKI from CKD as there are differences in platelet aggregation in people with AKI versus CKD.^8^ It is also important to consider the method of platelet function analysis, as there are differences in platelet aggregation measured by LTA and MEPA,^8^ which is hypothesized to be due to the lack of erythrocytes in the PRP used in LTA. It is difficult to compare our results with other studies in dogs, due to the difference in etiology of kidney injury, as well as the difference in platelet function testing.

We measured vWF:Ag and vWF:CBA to determine if vWF played a role in bleeding tendencies in dogs with AKI. As vWF:Ag in the AKI group was no different to the control group and was slightly higher than reported values,^16^, ^17^ our study confirms that it is not a decrease or deficiency in vWF concentrations which contributes to bleeding tendencies in dogs with AKI. Our study results more closely reflect an acquired type II vWD phenotype, as the AKI group had significantly higher vWF:Ag:vWF:CBA compared to the control group. Acquired von Willebrand syndrome (AVWS) occurs in people with uremia and is defined as an acquired bleeding disorder with clinical and laboratory features similar to inherited von Willebrand disease.^33^ Although AVWS occurs in many disease processes in people, the incidence of AVWS in uremic patients is rare, with only 15 cases reported so far, which accounts for only 4% of underlying causes of AVWS in total.^33^ The proposed mechanism of AVWS in people is due to increased proteolytic degradation of vWF by specific proteases.^33^, ^34^ This proposed mechanism has been supported by findings of low vWF ristocetin (vWF:RCo) to vWF:Ag ratio, which is another method for detecting decreased large MW vWF in people, as well as a lack of large MW vWF on multimeric analysis in CKD.^35^ However, factors other than vWF:Ag concentration or vWF multimeric pattern might play a role in bleeding tendencies in uremic people with CKD, as normal, increased, or decreased vWF:Ag, vWF:RCo, and multimeric pattern occurs with and without prolonged bleeding times.^35^, ^36^, ^37^ These variable results suggest that the underlying cause of bleeding tendencies is not completely understood, possibly being multifactorial or affected by other comorbidities or confounding factors.

Primary hemostatic disorder occurs in dogs with experimentally induced CKD, indicated by a prolongation in BMBT.^11^ However, the underlying mechanism is unknown as there are increased vWF:Ag concentrations, no change in multimeric distribution of vWF,^11^ and normal AA‐, ADP‐, and COL‐induced LTA.^10^ This is contrary to our findings which characterized primary hemostatic disorders in naturally occurring AKI with decreased COL‐induced platelet aggregometry, and abnormal binding of vWF to COL, as indicated by the high vWF:Ag:vWF:CBA ratio. The difference in our findings is likely associated with the different nature of disease, as well as the different methods in primary hemostatic assays, though we cannot exclude other causes of primary hemostatic dysfunction not measured in our study.

Buccal mucosal bleeding time was used to evaluate primary hemostatic function as it is readily available to most veterinary practitioners. Our study found prolonged BMBT in dogs with naturally occurring AKI, compared to known reference intervals of 1.7 to 4.8 minutes.^18^, ^19^ As we did not compare BMBT to the control group under the guidance of our ethics committee, we performed Spearman's rank test to measure correlation between BMBT and other measured variables. There was no correlation between BMBT and other primary hemostatic function tests, contrary to a negative correlation between vWF:Ag and BMBT in dogs with type I‐III vWD and dogs with thrombocytopathia.^38^ Uremic dogs can have normal^39^ or prolonged BMBTs, with mean (SD) of 12.60 (±6.05) minutes,^19^ and 7.0 (±0.4) minutes.^11^ These differences in BMBT might be due to the etiology of uremia or known variability in the BMBT measurement technique.^19^, ^40^ Interobserver variability was minimized in our study by having only 2 of the authors performing the test, although intraobserver variability would have been present.^40^ Buccal mucosal bleeding time might have been longer if the platelet count in our study population was no different to the control group, and the test procedure was not discontinued in the dog which had BMBT of 10 minutes or greater. We conclude that either BMBT is not appropriate for measuring primary hemostatic function in dogs with AKI due to these variabilities or perhaps there are other mechanisms of primary hemostatic dysfunction not measured in our study which caused the prolonged BMBT.^10^, ^11^

There are several limitations to our study. The AKI group had lower AA‐ and ADP‐induced platelet aggregation compared to the control group which was not statistically significant. As this is a pilot study, the small sample size might have contributed to a type II error. A sample size of 26 dogs in each group would be required to detect a difference in platelet aggregation between the 2 groups, should such a difference exist. The small sample size might have also contributed to a type II error in vWF:Ag concentrations, in which 115 dogs would be required to detect a difference between the 2 groups, should such a difference exist. There were many variables with strong correlation which did not reach significance, in which 24 dogs would be required in each group to reach significant difference if present. In addition, as already discussed, the difference in PCV and platelet count might have also influenced our results. If PCV and platelet count were standardized between the 2 groups, there might be decreased or increased difference in platelet aggregation respectively.

We aimed to characterize platelet function in dogs with AKI; however, we also included dogs with AoCKD. This inclusion of dogs with CKD might limit our ability to purely state that the findings are specific to AKI, as primary hemostatic function might have been contributed from mechanisms associated with CKD also. Although our study design excluded known causes of AKI, other unknown comorbidities might have influenced the results.

In conclusion, we found that dogs with AKI have abnormal primary hemostatic function, with decreased COL‐induced MEPA and abnormal vWF:CBA. Buccal mucosal bleeding time is not an appropriate test to determine if primary hemostatic dysfunctions are present in dogs with AKI. This study did not determine if primary hemostatic dysfunctions found in this study are associated with clinical bleeding.

## CONFLICT OF INTEREST DECLARATION

Authors declare no conflict of interest.

## OFF‐LABEL ANTIMICROBIAL DECLARATION

Authors declare no off‐label use of antimicrobials.

## INSTITUTIONAL ANIMAL CARE AND USE COMMITTEE (IACUC) OR OTHER APPROVAL DECLARATION

This study was approved by the Royal Veterinary College Clinical Research Ethical Review Board.

## HUMAN ETHICS APPROVAL DECLARATION

Authors declare human ethics approval was not needed for this study.

## References

[1] Visconti L , Cernaro V , Ricciardi CA , et al. Renal biopsy: still a landmark for the nephrologist. World J Nephrol. 2016;5:321‐327. 27458561 10.5527/wjn.v5.i4.321 PMC4936339

[2] Segev G , Langston C , Takada K , Kass PH , Cowgill DL . Validation of a clinical scoring system for outcome prediction in dogs with acute kidney injury managed by hemodialysis. J Vet Intern Med. 2016;30:803‐807. 26995335 10.1111/jvim.13930 PMC4913579

[3] Acierno MJ . Continuous renal replacement therapy in dogs and cats. Vet Clin North Am Small Anim Pract. 2011;41:135‐146. 21251514 10.1016/j.cvsm.2010.09.001

[4] Elliott DA . Nutritional considerations for the dialytic patient. Vet Clin North Am Small Anim Pract. 2011;41:239‐250. 21251520 10.1016/j.cvsm.2010.10.001

[5] Casari C , Bergmeier W . Acquired platelet disorders. Thromb Res. 2016;141:S73‐S75. 27207431 10.1016/S0049-3848(16)30371-1

[6] Kaw D , Malhotra D . Platelet dysfunction and end‐stage renal disease. Semin Dial. 2006;19:317‐322. 16893410 10.1111/j.1525-139X.2006.00179.x

[7] Sohal AS , Gangji AS , Crowther MA , Treleaven D . Uremic bleeding: pathophysiology and clinical risk factors. Thromb Res. 2006;118:417‐422. 15993929 10.1016/j.thromres.2005.03.032

[8] Malyszko J , Maluszko JS , Pawlak D , et al. Hemostasis, platelet function and serotonin in acute and chronic renal failure. Thromb Res. 1996;83:351‐361. 8873344 10.1016/0049-3848(96)00145-4

[9] Ho SJ , Gemmell R , Brighton T . Platelet function testing in uraemic patients. Hematology. 2008;13:49‐58. 18534067 10.1179/102453308X315834

[10] Brassard JA , Meyers KM , Person M , Dhein C . Experimentally induced renal failure in the dog as an animal model of uremic bleeding. J Lab Clin Med. 1994;124:48‐54. 8035102

[11] Brassard JA , Meyers K . von Willebrand factor is not altered in azotemic dogs with prolonged bleeding time. J Lab Clin Med. 1994;124:55‐62. 8035104

[12] Forsythe LT , Jackson ML , Meric SM . Whole blood platelet aggregation in uremic dogs. Am J Vet Res. 1989;50:1754‐1757. 2802308

[13] Dudley A , Byron JK , Jo M . Comparison of platelet function and viscoelastic test results between healthy dogs and dogs with naturally occurring chronic kidney disease. Am J Vet Res. 2017;78:589‐600. 28441050 10.2460/ajvr.78.5.589

[14] Li RHL , Chan DL . Evaluation of platelet function using multiple electrode platelet aggregometry in dogs with septic peritonitis. J Vet Emerg Crit Care. 2016;26:630‐638. 10.1111/vec.12508 27428542

[15] Friederichs KR , Harr KE , Freeman KP , et al. ASVCP reference interval guidelines: determination of de novo reference intervals in veterinary species and other related topics. Vet Clin Pathol. 2012;41:441‐453. 23240820 10.1111/vcp.12006

[16] Johnstone IB . Plasma von Willebrand factor‐collagen binding activity in normal dogs and in dogs with von Willebrand's disease. J Vet Diagn Invest. 1999;11:308‐313. 10424644 10.1177/104063879901100402

[17] Sabino EP , Erb HN , Catalfamo JL . Development of a collagen‐binding activity assay as a screening test for type II von Willebrand disease in dogs. Am J Vet Res. 2006;67:242‐249. 16454628 10.2460/ajvr.67.2.242

[18] Forsythe LT , Willis SE . Evaluating oral mucosa bleeding times in healthy dogs using a spring‐loaded device. Can Vet J. 1989;30:344‐345. 17423293 PMC1681208

[19] Jergens A , Turrentine MA , Kraus KH , Johnson GS . Buccal mucosal bleeding times of healthy dogs and of dogs in various pathological states, including thrombocytopenia, uremia, and von Willebrand's disease. Am J Vet Res. 1987;48:1337‐1342. 3499100

[20] Goggs R , Poole AW . State of the art review platelet signaling—a primer. J Vet Emerg Crit Care. 2012;22:5‐29. 10.1111/j.1476-4431.2011.00704.x 22316389

[21] Wardrop KJ , Dhein CR , Frenier S , Meyers K . Altered hemostasis in a dog with chronic renal failure. J Am Anim Hosp Assoc. 1989;25:325‐329.

[22] Jandrey KE . Assessment of platelet function. J Vet Emerg Crit Care. 2012;22(1):81‐98. 10.1111/j.1476-4431.2011.00707.x 23016745

[23] Kalbantner K , Baumgarten A , Mischke R . Measurement of platelet function in dogs using a novel impedance aggregometer. Vet J. 2010;185:144‐151. 19879171 10.1016/j.tvjl.2009.05.028

[24] Marschner CB , Kristensen AT , Spodsberg EH , Wiinber B . Evaluation of platelet aggregometry in dogs using multiplate platelet analyzer: impact of anticoagulant choice and assay duration. J Vet Emerg Crit Care. 2012;22:107‐115. 10.1111/j.1476-4431.2011.00709.x 23016746

[25] Lynch AM , deLaforcade AM , Meola D , et al. Assessment of hemostatic changes in a model of acute hemorrhage in dogs. J Vet Emerg Crit Care. 2016;26:333‐343. 10.1111/vec.12457 26890726

[26] Gawaz MP , Dobos G , Spath M , et al. Impaired function of platelet membrane glycoprotein IIb‐IIIa in end‐stage renal disease. J Am Soc Nephrol. 1994;5:36‐46. 7524718 10.1681/ASN.V5136

[27] Kuiper GJAJM , Houben R , Wetzels RJH , et al. The use of regression analysis in determining reference intervals for low hematocrit and thrombocyte count in multiple electrode aggregometry and platelet function analyzer 100 testing of platelet function. Platelets. 2017;9:1‐8. 10.1080/09537104.2016.1257782 28067094

[28] Bochsen L , Johansson PI , Kristensen AT , Daugaard G , Ostrowski SR . The influence of platelets, plasma and red blood cells on functional haemostatic assays. Blood Coagul Fibrinolysis. 2011;22:167‐175. 21330915 10.1097/MBC.0b013e3283424911

[29] Hanke AA , Roberg K , Monoca E , et al. Impact of platelet count on results obtained from multiple electrode platelet aggregometry (Multiplate™). Eur J Med Res. 2010;15:214‐219. 20562061 10.1186/2047-783X-15-5-214 PMC3352011

[30] Femia EA , Scavone M , Lecchi A , Cattaneo M . Effect of platelet count on platelet aggregation measured with impedance aggregometry (Multiplate™ analyzer) and with light transmission aggregometry. J Thromb Haemost. 2013;11:2193‐2196. 24148217 10.1111/jth.12432

[31] Muller MR , Salat A , Pulaki S , et al. Influence of hematocrit and platelet count on impedance and reactivity of whole blood for electrical aggregometry. J Pharmacol Toxicol Methods. 1995;23:17‐22. 10.1016/1056-8719(94)00075-f 7496042

[32] Wurtz M , Hvas AM , Kristensen SD , Grove E . Platelet aggregation is dependent on platelet count in patients with coronary artery disease. Thromb Res. 2012;129:56‐61. 21917303 10.1016/j.thromres.2011.08.019

[33] Federici AB , Budde U , Castaman G , Rand JH , Tiede A . Current diagnostic and therapeutic approaches to patients with acquired von Willebrand syndrome: a 2013 update. Semin Thromb Hemost. 2013;39:191‐201. 23397553 10.1055/s-0033-1334867

[34] Rodeghiero F , Castaman G , Lombardi R , Mannucci P . Von Willebrand factor abnormalities in two patients with uraemia. Lancet. 1988;1:1016‐1017. 10.1016/s0140-6736(88)90762-3 2902444

[35] Gralnick HR , McKeown LP , Williams SB , et al. Plasma and platelet von Willebrand factor defects in uremia. Am J Med. 1988;85:806‐810. 3264111 10.1016/s0002-9343(88)80025-1

[36] Castaman G , Rodeghiero F , Lattuada A , la Greca G , Mannucci PM . Multimeric pattern of plasma and platelet von Willebrand factor is normal in uremic patients. Am J Hematol. 1993;44:266‐269. 8237998 10.1002/ajh.2830440409

[37] Casonato A , Pntara E , Vertolli UP , et al. Plasma and platelet von Willebrand factor abnormalities in patients with uremia: lack of correlation with uremic bleeding. Clin Appl Thromb Hemost. 2001;7:81‐86. 11292197 10.1177/107602960100700201

[38] Brooks M , Catalfamo J . Buccal mucosal bleeding time is prolonged in canine models of primary hemostatic disorders. Thromb Haemost. 1993;70:777‐780. 8128434

[39] Polzin DJ , Osborne CA , Hayden D . Influence of reduced protein diets on morbidity, mortality, and renal function in dogs with induced chronic renal failure. Am J Vet Res. 1983;45:506‐517. 6711979

[40] Sato I , Anderson GA , Parry B . An interobserver and intraobserver study of buccal mucosal bleeding time in greyhounds. Res Vet Sci. 2000;68:41‐45. 10684757 10.1053/rvsc.1999.0334

